# Supplementary material for: The multiple roles of lipid metabolism in yeast physiology during beer fermentation
Source: Genet Mol Biol. 2022 Sep 16;45(3):e20210325. doi: 10.1590/1678-4685-GMB-2021-0325 (PMC9511687; doi:10.1590/1678-4685-GMB-2021-0325)
Supplement: Figure S1 - [file 1415-4757-GMB-45-3-e20210325-s1.pdf]

Genetics and Molecular Biology

https://doi.org/10.1590/1678-4685-GMB-2021-0325

Supplementary Material to "The multiple roles of lipid metabolism in yeast physiology during beer fermentation"

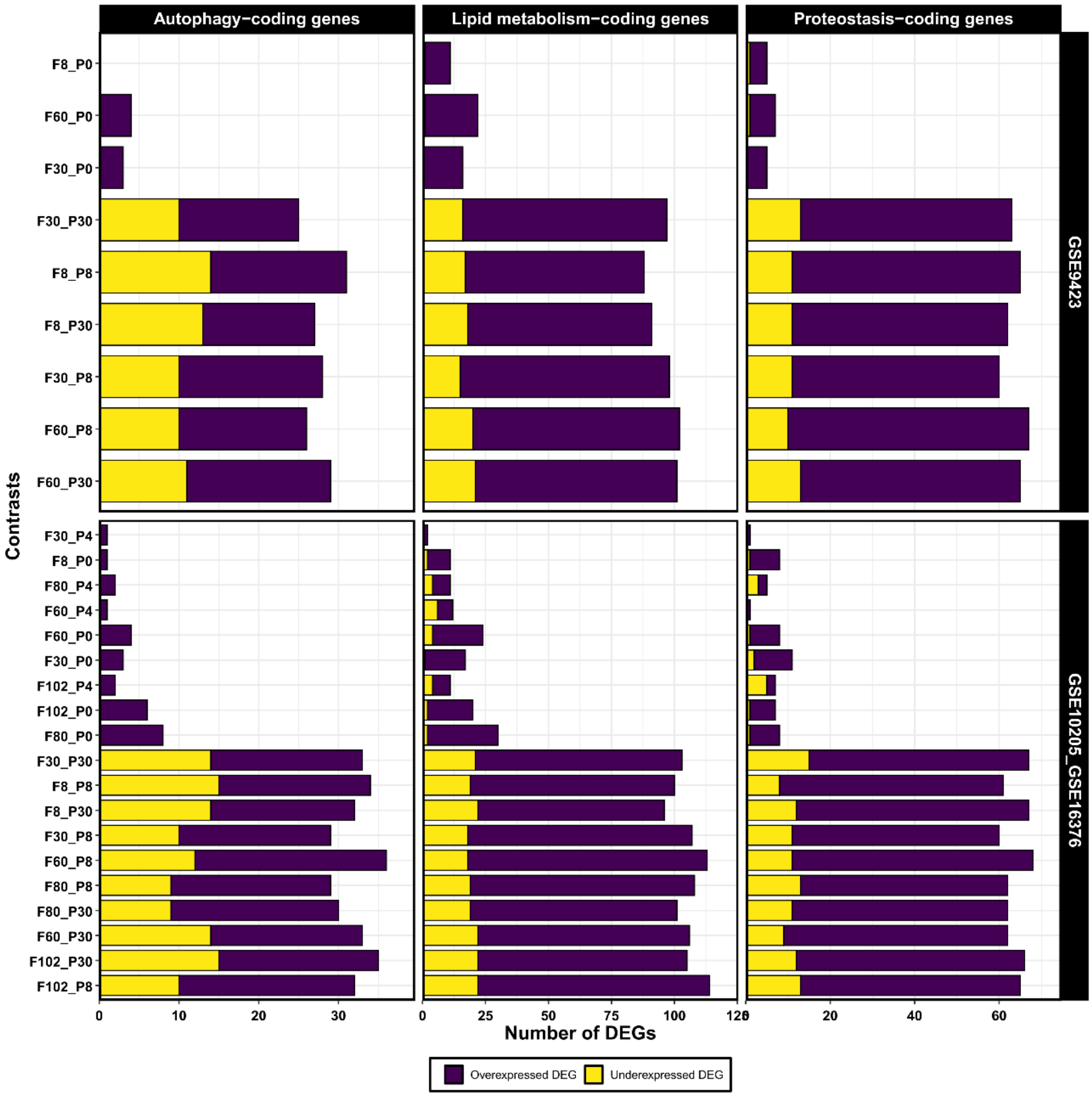

Figure S1 - Frequency of total downregulated and upregulated genes associated with autophagy, lipid metabolism, and proteostasis (ALP) in different time point comparisons in the transcriptome single-analysis (GSE9423) and meta-analysis (GSE10205 versus GSE16376). F: beer fermentation; P: yeast biomass propagation.
