# Supplementary material for: The multiple roles of lipid metabolism in yeast physiology during beer fermentation
Source: Genet Mol Biol. 2022 Sep 16;45(3):e20210325. doi: 10.1590/1678-4685-GMB-2021-0325 (PMC9511687; doi:10.1590/1678-4685-GMB-2021-0325)
Supplement: Table S1 - [file 1415-4757-GMB-45-3-e20210325-s2.pdf]

Genetics and Molecular Biology

https://doi.org/10.1590/1678-4685-GMB-2021-0325

Supplementary Material to "The multiple roles of lipid metabolism in yeast physiology during beer fermentation"

Table S1 - DNA microarray gene expression datasets used in this work.

Source	GEO_sample_file	Sample_name	Sample	Organism	Strain	FTP_file
GSE9423	GSM239499	Fermentation 30 hours	Ferm_30h_B	Saccharomyces pastorianus	CB11	ftp://ftp.ncbi.nlm.nih.gov/geo/samples/GSM239nnn/GSM239499/suppl/GSMGSM239499.CEL.gz
GSE9423	GSM239503	Fermentation 60 hours	Ferm_60h_C	Saccharomyces pastorianus	CB11	ftp://ftp.ncbi.nlm.nih.gov/geo/samples/GSM239nnn/GSM239503/suppl/GSMGSM239503.CEL.gz
GSE9423	GSM239512	Propagation 30 hours	Prop_30h_C	Saccharomyces pastorianus	CB11	ftp://ftp.ncbi.nlm.nih.gov/geo/samples/GSM239nnn/GSM239512/suppl/GSMGSM239512.CEL.gz
GSE9423	GSM239504	Fermentation 8 hours	Ferm_8h_A	Saccharomyces pastorianus	CB11	ftp://ftp.ncbi.nlm.nih.gov/geo/samples/GSM239nnn/GSM239504/suppl/GSMGSM239504.CEL.gz
GSE9423	GSM239514	Propagation 8 hours	Prop_8h_B	Saccharomyces pastorianus	CB11	ftp://ftp.ncbi.nlm.nih.gov/geo/samples/GSM239nnn/GSM239514/suppl/GSMGSM239514.CEL.gz
GSE9423	GSM239501	Fermentation 60 hours	Ferm_60h_A	Saccharomyces pastorianus	CB11	ftp://ftp.ncbi.nlm.nih.gov/geo/samples/GSM239nnn/GSM239501/suppl/GSMGSM239501.CEL.gz

Source	GEO_sample_file	Sample_name	Sample	Organism	Strain	FTP_file
GSE9423	GSM239510	Propagation 30 hours	Prop_30h_A	Saccharomyces pastorianus	CB11	ftp://ftp.ncbi.nlm.nih.gov/geo/samples/GSM239nnn/GSM239510/suppl/GSMGSM239510.CEL.gz
GSE9423	GSM239515	Propagation 8 hours	Prop_8h_C	Saccharomyces pastorianus	CB11	ftp://ftp.ncbi.nlm.nih.gov/geo/samples/GSM239nnn/GSM239515/suppl/GSMGSM239515.CEL.gz
GSE9423	GSM239506	Fermentation 8 hours	Ferm_8h_C	Saccharomyces pastorianus	CB11	ftp://ftp.ncbi.nlm.nih.gov/geo/samples/GSM239nnn/GSM239506/suppl/GSMGSM239506.CEL.gz
GSE9423	GSM239505	Fermentation 8 hours	Ferm_8h_B	Saccharomyces pastorianus	CB11	ftp://ftp.ncbi.nlm.nih.gov/geo/samples/GSM239nnn/GSM239505/suppl/GSMGSM239505.CEL.gz
GSE9423	GSM239511	Propagation 30 hours	Prop_30h_B	Saccharomyces pastorianus	CB11	ftp://ftp.ncbi.nlm.nih.gov/geo/samples/GSM239nnn/GSM239511/suppl/GSMGSM239511.CEL.gz
GSE9423	GSM239502	Fermentation 60 hours	Ferm_60h_B	Saccharomyces pastorianus	CB11	ftp://ftp.ncbi.nlm.nih.gov/geo/samples/GSM239nnn/GSM239502/suppl/GSMGSM239502.CEL.gz
GSE9423	GSM239508	Propagation 0 hours	Prop_0h_B	Saccharomyces pastorianus	CB11	ftp://ftp.ncbi.nlm.nih.gov/geo/samples/GSM239nnn/GSM239508/suppl/GSMGSM239508.CEL.gz
GSE9423	GSM239500	Fermentation 30 hours	Ferm_30h_C	Saccharomyces pastorianus	CB11	ftp://ftp.ncbi.nlm.nih.gov/geo/samples/GSM239nnn/GSM239500/suppl/GSMGSM239500.CEL.gz
GSE9423	GSM239507	Propagation 0 hours	Prop_0h_A	Saccharomyces pastorianus	CB11	ftp://ftp.ncbi.nlm.nih.gov/geo/samples/GSM239nnn/GSM239507/suppl/GSMGSM239507.CEL.gz
GSE9423	GSM239509	Propagation 0 hours	Prop_0h_C	Saccharomyces pastorianus	CB11	ftp://ftp.ncbi.nlm.nih.gov/geo/samples/GSM239nnn/GSM239509/suppl/GSMGSM239509.CEL.gz
GSE9423	GSM239513	Propagation 8 hours	Prop_8h_A	Saccharomyces pastorianus	CB11	ftp://ftp.ncbi.nlm.nih.gov/geo/samples/GSM239nnn/GSM239513/suppl/GSMGSM239513.CEL.gz

Source	GEO_sample_file	Sample_name	Sample	Organism	Strain	FTP_file
GSE10205	GSM257787	Fermentation 102 hours	Ferm_102h_A	Saccharomyces pastorianus	CB11	ftp://ftp.ncbi.nlm.nih.gov/geo/samples/GSM257nnn/GSM257787/suppl/GSMGSM257787.CEL.gz
GSE10205	GSM257776	Fermentation 8 hours	Ferm_8h_A	Saccharomyces pastorianus	CB11	ftp://ftp.ncbi.nlm.nih.gov/geo/samples/GSM257nnn/GSM257776/suppl/GSMGSM257776.CEL.gz
GSE10205	GSM257778	Fermentation 8 hours	Ferm_8h_C	Saccharomyces pastorianus	CB11	ftp://ftp.ncbi.nlm.nih.gov/geo/samples/GSM257nnn/GSM257778/suppl/GSMGSM257778.CEL.gz
GSE10205	GSM257789	Fermentation 102 hours	Ferm_102h_C	Saccharomyces pastorianus	CB11	ftp://ftp.ncbi.nlm.nih.gov/geo/samples/GSM257nnn/GSM257789/suppl/GSMGSM257789.CEL.gz
GSE10205	GSM257780	Fermentation 30 hours	Ferm_30h_B	Saccharomyces pastorianus	CB11	ftp://ftp.ncbi.nlm.nih.gov/geo/samples/GSM257nnn/GSM257780/suppl/GSMGSM257780.CEL.gz
GSE10205	GSM257782	Fermentation 60 hours	Ferm_60 h_B	Saccharomyces pastorianus	CB11	ftp://ftp.ncbi.nlm.nih.gov/geo/samples/GSM257nnn/GSM257782/suppl/GSMGSM257782.CEL.gz
GSE10205	GSM257786	Fermentation 80 hours	Ferm_80h_C	Saccharomyces pastorianus	CB11	ftp://ftp.ncbi.nlm.nih.gov/geo/samples/GSM257nnn/GSM257786/suppl/GSMGSM257786.CEL.gz
GSE10205	GSM257785	Fermentation 80 hours	Ferm_80h_B	Saccharomyces pastorianus	CB11	ftp://ftp.ncbi.nlm.nih.gov/geo/samples/GSM257nnn/GSM257785/suppl/GSMGSM257785.CEL.gz
GSE10205	GSM257781	Fermentation 60 hours	Ferm_60h_A	Saccharomyces pastorianus	CB11	ftp://ftp.ncbi.nlm.nih.gov/geo/samples/GSM257nnn/GSM257781/suppl/GSMGSM257781.CEL.gz
GSE10205	GSM257783	Fermentation 60 hours	Ferm_60h_C	Saccharomyces pastorianus	CB11	ftp://ftp.ncbi.nlm.nih.gov/geo/samples/GSM257nnn/GSM257783/suppl/GSMGSM257783.CEL.gz
GSE10205	GSM257784	Fermentation 80 hours	Ferm_80h_A	Saccharomyces pastorianus	CB11	ftp://ftp.ncbi.nlm.nih.gov/geo/samples/GSM257nnn/GSM257784/suppl/GSMGSM257784.CEL.gz

Source	GEO_sample_file	Sample_name	Sample	Organism	Strain	FTP_file
GSE10205	GSM257777	Fermentation 8 hours	Ferm_8h_B	Saccharomyces pastorianus	CB11	ftp://ftp.ncbi.nlm.nih.gov/geo/samples/GSM257nnn/GSM257777/suppl/GSMGSM257777.CEL.gz
GSE10205	GSM257779	Fermentation 30 hours	Ferm_30h_A	Saccharomyces pastorianus	CB11	ftp://ftp.ncbi.nlm.nih.gov/geo/samples/GSM257nnn/GSM257779/suppl/GSMGSM257779.CEL.gz
GSE10205	GSM257788	Fermentation 102 hours	Ferm_102h_B	Saccharomyces pastorianus	CB11	ftp://ftp.ncbi.nlm.nih.gov/geo/samples/GSM257nnn/GSM257788/suppl/GSMGSM257788.CEL.gz
GSE16376	GSM410831	Propagation 0 hours	Prop_0h_A	Saccharomyces pastorianus	CB11	ftp://ftp.ncbi.nlm.nih.gov/geo/samples/GSM410nnn/GSM410831/suppl/GSMGSM410831.CEL.gz
GSE16376	GSM410832	Propagation 0 hours	Prop_0h_B	Saccharomyces pastorianus	CB11	ftp://ftp.ncbi.nlm.nih.gov/geo/samples/GSM410nnn/GSM410832/suppl/GSMGSM410832.CEL.gz
GSE16376	GSM410833	Propagation 0 hours	Prop_0h_C	Saccharomyces pastorianus	CB11	ftp://ftp.ncbi.nlm.nih.gov/geo/samples/GSM410nnn/GSM410833/suppl/GSMGSM410833.CEL.gz
GSE16376	GSM410834	Propagation 4 hours	Prop_4h_A	Saccharomyces pastorianus	CB11	ftp://ftp.ncbi.nlm.nih.gov/geo/samples/GSM410nnn/GSM410834/suppl/GSMGSM410834.CEL.gz
GSE16376	GSM410835	Propagation 4 hours	Prop_4h_B	Saccharomyces pastorianus	CB11	ftp://ftp.ncbi.nlm.nih.gov/geo/samples/GSM410nnn/GSM410835/suppl/GSMGSM410835.CEL.gz
GSE16376	GSM410836	Propagation 4 hours	Prop_4h_C	Saccharomyces pastorianus	CB11	ftp://ftp.ncbi.nlm.nih.gov/geo/samples/GSM410nnn/GSM410836/suppl/GSMGSM410836.CEL.gz
GSE16376	GSM410837	Propagation 8 hours	Prop_8h_A	Saccharomyces pastorianus	CB11	ftp://ftp.ncbi.nlm.nih.gov/geo/samples/GSM410nnn/GSM410837/suppl/GSMGSM410837.CEL.gz
GSE16376	GSM410838	Propagation 8 hours	Prop_8h_B	Saccharomyces pastorianus	CB11	ftp://ftp.ncbi.nlm.nih.gov/geo/samples/GSM410nnn/GSM410838/suppl/GSMGSM410838.CEL.gz

Source	GEO_sample_file	Sample_name	Sample	Organism	Strain	FTP_file
GSE16376	GSM410839	Propagation 8 hours	Prop_8h_C	Saccharomyces pastorianus	CB11	ftp://ftp.ncbi.nlm.nih.gov/geo/samples/GSM410nnn/GSM410839/suppl/GSMGSM410839.CEL.gz
GSE16376	GSM410840	Propagation 30 hours	Prop_30h_A	Saccharomyces pastorianus	CB11	ftp://ftp.ncbi.nlm.nih.gov/geo/samples/GSM410nnn/GSM410840/suppl/GSMGSM410840.CEL.gz
GSE16376	GSM410841	Propagation 30 hours	Prop_30h_B	Saccharomyces pastorianus	CB11	ftp://ftp.ncbi.nlm.nih.gov/geo/samples/GSM410nnn/GSM410841/suppl/GSMGSM410841.CEL.gz
GSE16376	GSM410842	Propagation 30 hours	Prop_30h_C	Saccharomyces pastorianus	CB11	ftp://ftp.ncbi.nlm.nih.gov/geo/samples/GSM410nnn/GSM410842/suppl/GSMGSM410842.CEL.gz
