# Supplementary material for: The multiple roles of lipid metabolism in yeast physiology during beer fermentation
Source: Genet Mol Biol. 2022 Sep 16;45(3):e20210325. doi: 10.1590/1678-4685-GMB-2021-0325 (PMC9511687; doi:10.1590/1678-4685-GMB-2021-0325)
Supplement: Table S2 - [file 1415-4757-GMB-45-3-e20210325-s3.pdf]

Genetics and Molecular Biology

https://doi.org/10.1590/1678-4685-GMB-2021-0325

Supplementary Material to “The multiple roles of lipid metabolism in yeast physiology during beer fermentation”

Table S2 - Upregulated ALP Pan-DEGs associated with lipid droplets.

Feature	Meta logFC	SE	SD	Genes	Gene type	Betweenness	Degree	Node type	Cluster number
ACH1	2.60	0.0666	0.0941	YBL015W	Lipid metabolism-coding genes	380.97	19	Non-Hub-Non-Bottleneck	Cluster 9
CPR5	5.24	0.176	0.249	YDR304C	Proteostasis-coding genes	740.91	6	Non-Hub-Non-Bottleneck	Cluster 15
DPL1	2.30	0.163	0.231	YDR294C	Lipid metabolism-coding genes	849.80	14	Non-Hub-Non-Bottleneck	Cluster 27
ERG1	2.30	0.237	0.335	YGR175C	Lipid metabolism-coding genes	736.97	12	Non-Hub-Non-Bottleneck	Cluster 31

Feature	Meta logFC	SE	SD	Genes	Gene type	Betweenness	Degree	Node type	Cluster number
ERG27	4.05	0.0784	0.111	YLR100W	Lipid metabolism-coding genes	439.15	15	Non-Hub-Non-Bottleneck	Cluster 31
ERG6	3.77	0.0132	0.0186	YML008C	Lipid metabolism-coding genes	6353.53	34	Hub-Bottleneck	Cluster 31
FAA4	3.44	0.92	1.3	YMR246W	Lipid metabolism-coding genes	1317.20	17	Non-Hub-Non-Bottleneck	Cluster 1
FAS1	2.52	0.101	0.143	YKL182W	Lipid metabolism-coding genes	3356.28	20	Non-Hub-Bottleneck	Cluster 13
GPT2	2.28	0.00921	0.013	YKR067W	Lipid metabolism-coding genes	561.95	17	Non-Hub-Non-Bottleneck	Cluster 1
HFD1	2.62	0.0685	0.0969	YMR110C	Lipid metabolism-coding genes	6383.51	41	Hub-Bottleneck	Cluster 9
KAR2	2.79	0.172	0.243	YJL034W	Proteostasis-coding genes	3747.57	31	Hub-Bottleneck	Cluster 6
PDI1	2.93	0.112	0.159	YCL043C	Proteostasis-coding genes	3019.99	25	Non-Hub-Bottleneck	Cluster 2
PMT1	2.53	0.048	0.0679	YDL095W	Proteostasis-coding genes	1938.03	25	Non-Hub-Bottleneck	Cluster 29

Feature	Meta logFC	SE	SD	Genes	Gene type	Betweenness	Degree	Node type	Cluster number
POX1	2.52	0.0107	0.0151	YGL205W	Lipid metabolism-coding genes	4000.36	28	Hub-Bottleneck	Cluster 2
RPL5	3.28	0.331	0.468	YPL131W	Proteostasis-coding genes	1977.12	105	Hub-Bottleneck	Cluster 11
SSA1	3.60	0.346	0.489	YAL005C	Proteostasis-coding genes	7008.87	56	Hub-Bottleneck	Cluster 4
YJU3	2.89	0.315	0.446	YKL094W	Lipid metabolism-coding genes	480.35	14	Non-Hub-Non-Bottleneck	Cluster 1
