# Supplementary material for: The multiple roles of lipid metabolism in yeast physiology during beer fermentation
Source: Genet Mol Biol. 2022 Sep 16;45(3):e20210325. doi: 10.1590/1678-4685-GMB-2021-0325 (PMC9511687; doi:10.1590/1678-4685-GMB-2021-0325)
Supplement: Table S3 - [file 1415-4757-GMB-45-3-e20210325-s4.pdf]

Genetics and Molecular Biology

https://doi.org/10.1590/1678-4685-GMB-2021-0325

Supplementary Material to "The multiple roles of lipid metabolism in yeast physiology during beer fermentation"

Table S3 - Transcriptome and proteolipidome data of nodes associated with lipid droplet-linked protein (LDP) network.

Feature	Associated molecules	Mean FC 12 h versus 6 h	Mean FC 20 h versus 6 h	Meta logFC	Node type abbreviation	Cluster	Condition	Expression data type	SE	SD
POT1	Fatty esters	4.00	19.75	3.21	HB	Cluster 2	Lipid metabolism-coding genes	Expressed in proteome and transcriptome	0.139	0.197
LCB5	Sphingomyelins	1.08	1.31	NA	NA	NA	NA	Expressed in proteome	NA	NA
OLE1	Fatty esters	0.22	0.28	2.12	HB	Cluster 4	Lipid metabolism-coding genes	Expressed in proteome and transcriptome	0.00691	0.00978
ERG3	Sterols	0.22	0.22	3.74	NHNB	Cluster 31	Lipid metabolism-coding genes	Expressed in proteome and transcriptome	0.0404	0.0571
ERG6	Sterols	0.85	0.77	3.77	HB	Cluster 31	Lipid metabolism-coding genes	Expressed in proteome and transcriptome	0.0132	0.0186

Feature	Associated molecules	Mean FC 12 h versus 6 h	Mean FC 20 h versus 6 h	Meta logFC	Node type abbreviation	Cluster	Condition	Expression data type	SE	SD
ERG2	Sterols	0.85	0.73	2.85	NHB	Cluster 31	Lipid metabolism-coding genes	Expressed in proteome and transcriptome	0.0615	0.0869
ERG26	Sterols	1.44	1.32	4.17	NHNB	Cluster 31	Lipid metabolism-coding genes	Expressed in proteome and transcriptome	0.011	0.0155
ERG27	Sterols	0.84	0.55	4.05	NHNB	Cluster 31	Lipid metabolism-coding genes	Expressed in proteome and transcriptome	0.0784	0.111
POX1	Fatty esters	1.01	9.81	2.52	HB	Cluster 2	Lipid metabolism-coding genes	Expressed in proteome and transcriptome	0.0107	0.0151
LSC2	Fatty esters	3.24	5.96	NA	NA	NA	NA	Expressed in proteome	NA	NA
TGL4	Glycerophosphoethanolamines	1.25	1.35	NA	NA	NA	NA	Expressed in proteome	NA	NA
FAA4	Fatty esters	0.42	0.40	3.44	NHNB	Cluster 1	Lipid metabolism-coding genes	Expressed in proteome and transcriptome	0.92	1.3
FAA1	Fatty esters	1.93	2.19	NA	NA	NA	NA	Expressed in proteome	NA	NA
SLC1	Fatty esters	0.92	0.89	NA	NA	NA	NA	Expressed in proteome	NA	NA

Feature	Associated molecules	Mean FC 12 h versus 6 h	Mean FC 20 h versus 6 h	Meta logFC	Node type abbreviation	Cluster	Condition	Expression data type	SE	SD
YJU3	Fatty esters	1.23	1.42	2.89	NHNB	Cluster 1	Lipid metabolism-coding genes	Expressed in proteome and transcriptome	0.315	0.446
GPT2	Fatty esters	2.50	4.15	2.28	NHNB	Cluster 1	Lipid metabolism-coding genes	Expressed in proteome and transcriptome	0.00921	0.013
TGL4	Fatty esters	1.25	1.35	NA	NA	NA	NA	Expressed in proteome	NA	NA
GPD2	Fatty esters	0.63	0.68	NA	NA	NA	NA	Expressed in proteome	NA	NA
LOA1	Fatty esters	NA	NA	NA	NA	NA	NA	No expression data	NA	NA
YJU3	Monoradylglycerols	1.23	1.42	2.89	NHNB	Cluster 1	Lipid metabolism-coding genes	Expressed in proteome and transcriptome	0.315	0.446
LSC2	Quinones and hydroquinones	3.24	5.96	NA	NA	NA	NA	Expressed in proteome	NA	NA
GPD2	Quinones and hydroquinones	0.63	0.68	NA	NA	NA	NA	Expressed in proteome	NA	NA
ALD4	Quinones and hydroquinones	1.77	6.96	2.30	HB	Cluster 9	Lipid metabolism-coding genes	Expressed in proteome and transcriptome	0.0698	0.0988
URE2	Fatty aldehydes	0.96	1.27	NA	NA	NA	NA	Expressed in proteome	NA	NA

Feature	Associated molecules	Mean FC 12 h versus 6 h	Mean FC 20 h versus 6 h	Meta logFC	Node type abbreviation	Cluster	Condition	Expression data type	SE	SD
FAA4	Fatty acids	0.42	0.40	3.44	NHNB	Cluster 1	Lipid metabolism-coding genes	Expressed in proteome and transcriptome	0.92	1.3
FAA1	Fatty acids	1.93	2.19	NA	NA	NA	NA	Expressed in proteome	NA	NA
LEU4	Fatty acids	1.01	1.64	NA	NA	NA	NA	Expressed in proteome	NA	NA
LEU4	Fatty acids	1.01	1.64	NA	NA	NA	NA	Expressed in proteome	NA	NA
LCB5	Sphingoid bases	1.08	1.31	NA	NA	NA	NA	Expressed in proteome	NA	NA
POX1	Fatty esters	1.01	9.81	2.52	HB	Cluster 2	Lipid metabolism-coding genes	Expressed in proteome and transcriptome	0.0107	0.0151
FAS1	Fatty esters	1.04	0.96	NA	NA	NA	NA	Expressed in proteome	NA	NA
LCB1	Fatty esters	0.80	0.80	NA	NA	NA	NA	Expressed in proteome	NA	NA
LRO1	Fatty esters	0.59	0.35	NA	NA	NA	NA	Expressed in proteome	NA	NA
HFA1	Fatty esters	NA	NA	NA	NA	NA	NA	No expression data	NA	NA

Feature	Associated molecules	Mean FC 12 h versus 6 h	Mean FC 20 h versus 6 h	Meta logFC	Node type abbreviation	Cluster	Condition	Expression data type	SE	SD
CIT1	TCA acids	2.80	9.85	2.41	HB	Cluster 3	Lipid metabolism-coding genes	Expressed in proteome and transcriptome	0.258	0.366
ERG1	Isoprenoids	1.08	0.62	2.30	NHNB	Cluster 31	Lipid metabolism-coding genes	Expressed in proteome and transcriptome	0.237	0.335
ERG7	Isoprenoids	0.81	0.83	NA	NA	NA	NA	Expressed in proteome	NA	NA
ERG9	Isoprenoids	1.28	1.15	NA	NA	NA	NA	Expressed in proteome	NA	NA
TGL4	Glycerophosphocholines	1.25	1.35	NA	NA	NA	NA	Expressed in proteome	NA	NA
LRO1	Glycerophosphocholines	0.59	0.35	NA	NA	NA	NA	Expressed in proteome	NA	NA
POX1	Flavins	1.01	9.81	2.52	HB	Cluster 2	Lipid metabolism-coding genes	Expressed in proteome and transcriptome	0.0107	0.0151
FAT1	Fatty acids	1.04	1.01	NA	NA	NA	NA	Expressed in proteome	NA	NA
SLC1	Fatty acids	0.92	0.89	NA	NA	NA	NA	Expressed in proteome	NA	NA
OLE1	Fatty acids	0.22	0.28	2.12	HB	Cluster 4	Lipid metabolism-coding genes	Expressed in proteome and transcriptome	0.00691	0.00978

Feature	Associated molecules	Mean FC 12 h versus 6 h	Mean FC 20 h versus 6 h	Meta logFC	Node type abbreviation	Cluster	Condition	Expression data type	SE	SD
POX1	Fatty acids	1.01	9.81	2.52	HB	Cluster 2	Lipid metabolism-coding genes	Expressed in proteome and transcriptome	0.0107	0.0151
YJU3	Fatty acids	1.23	1.42	2.89	NHNB	Cluster 1	Lipid metabolism-coding genes	Expressed in proteome and transcriptome	0.315	0.446
TGL4	Fatty acids	1.25	1.35	NA	NA	NA	NA	Expressed in proteome	NA	NA
LOA1	Fatty acids	NA	NA	NA	NA	NA	NA	No expression data	NA	NA
POT1	Fatty acids	4.00	19.75	3.21	HB	Cluster 2	Lipid metabolism-coding genes	Expressed in proteome and transcriptome	0.139	0.197
TGL4	Isoprenoids	1.25	1.35	NA	NA	NA	NA	Expressed in proteome	NA	NA
FAT1	Fatty esters	1.04	1.01	NA	NA	NA	NA	Expressed in proteome	NA	NA
POX1	Fatty esters	1.01	9.81	2.52	HB	Cluster 2	Lipid metabolism-coding genes	Expressed in proteome and transcriptome	0.0107	0.0151
LIP5	Fatty esters	0.90	1.14	NA	NA	NA	NA	Expressed in proteome	NA	NA
LSC2	TCA acids	3.24	5.96	NA	NA	NA	NA	Expressed in proteome	NA	NA

Feature	Associated molecules	Mean FC 12 h versus 6 h	Mean FC 20 h versus 6 h	Meta logFC	Node type abbreviation	Cluster	Condition	Expression data type	SE	SD
ERG4	Sterols	0.76	0.75	NA	NA	NA	NA	Expressed in proteome	NA	NA
TGL4	Organic phosphoric acids	1.25	1.35	NA	NA	NA	NA	Expressed in proteome	NA	NA
ROT2	Piperidines	0.98	0.98	NA	NA	NA	NA	Expressed in proteome	NA	NA
ALD4	Amino acids and peptides	1.77	6.96	2.30	HB	Cluster 9	Lipid metabolism-coding genes	Expressed in proteome and transcriptome	0.0698	0.0988
ERG24	Sterols	NA	NA	2.39	NHNB	Cluster 31	Lipid metabolism-coding genes	Expressed in transcriptome	0.316	0.447
HMG1	Fatty acids	0.70	0.67	4.37	NHNB	Cluster 4	Lipid metabolism-coding genes	Expressed in proteome and transcriptome	0.158	0.223
ALD4	Monosaccharides	1.77	6.96	2.30	HB	Cluster 9	Lipid metabolism-coding genes	Expressed in proteome and transcriptome	0.0698	0.0988
ERG27	Glycosyl compounds	0.84	0.55	4.05	NHNB	Cluster 31	Lipid metabolism-coding genes	Expressed in proteome and transcriptome	0.0784	0.111
ERG6	Glycosyl compounds	0.85	0.77	3.77	HB	Cluster 31	Lipid metabolism-coding genes	Expressed in proteome and transcriptome	0.0132	0.0186

Feature	Associated molecules	Mean FC 12 h versus 6 h	Mean FC 20 h versus 6 h	Meta logFC	Node type abbreviation	Cluster	Condition	Expression data type	SE	SD
ERG2	Glycosyl compounds	0.85	0.73	2.85	NHB	Cluster 31	Lipid metabolism-coding genes	Expressed in proteome and transcriptome	0.0615	0.0869
ERG1	Sterols	1.08	0.62	2.30	NHNB	Cluster 31	Lipid metabolism-coding genes	Expressed in proteome and transcriptome	0.237	0.335
ERG7	Sterols	0.81	0.83	NA	NA	NA	NA	Expressed in proteome	NA	NA
FAT1	Bile acids	1.04	1.01	NA	NA	NA	NA	Expressed in proteome	NA	NA
GPX2	Amino acids and peptides	1.02	0.77	NA	NA	NA	NA	Expressed in proteome	NA	NA
URE2	Amino acids and peptides	0.96	1.27	NA	NA	NA	NA	Expressed in proteome	NA	NA
ERG10	Fatty esters	0.91	0.86	4.92	NHNB	Cluster 9	Lipid metabolism-coding genes	Expressed in proteome and transcriptome	0.0343	0.0485
DPL1	Sphingoid bases	1.13	1.23	2.30	NHNB	Cluster 6	Lipid metabolism-coding genes	Expressed in proteome and transcriptome	0.163	0.231
SUR2	Sphingoid bases	0.94	1.06	NA	NA	NA	NA	Expressed in proteome	NA	NA
LCB5	Sphingoid bases	1.08	1.31	NA	NA	NA	NA	Expressed in proteome	NA	NA

Feature	Associated molecules	Mean FC 12 h versus 6 h	Mean FC 20 h versus 6 h	Meta logFC	Node type abbreviation	Cluster	Condition	Expression data type	SE	SD
ADH7	Monosaccharides	NA	NA	NA	NA	NA	NA	No expression data	NA	NA
ACS1	Fatty esters	1.04	20.59	NA	NA	NA	NA	Expressed in proteome	NA	NA
PAH1	Fatty esters	0.96	1.35	NA	NA	NA	NA	Expressed in proteome	NA	NA
LCB1	Sphingoid bases	0.80	0.80	NA	NA	NA	NA	Expressed in proteome	NA	NA
HFA1	Amino acids and peptides	NA	NA	NA	NA	NA	NA	No expression data	NA	NA
ERG6	Glycosyl compounds	0.85	0.77	3.77	HB	Cluster 31	Lipid metabolism-coding genes	Expressed in proteome and transcriptome	0.0132	0.0186
LIP5	Glycosyl compounds	0.90	1.14	NA	NA	NA	NA	Expressed in proteome	NA	NA
GLT1	Amino acids and peptides	0.86	0.88	NA	NA	NA	NA	Expressed in proteome	NA	NA
FAT1	Isoprenoids	1.04	1.01	NA	NA	NA	NA	Expressed in proteome	NA	NA
SPO14	Pyrimidines	0.65	0.91	NA	NA	NA	NA	Expressed in proteome	NA	NA
LIP5	Amino acids and peptides	0.90	1.14	NA	NA	NA	NA	Expressed in proteome	NA	NA

Feature	Associated molecules	Mean FC 12 h versus 6 h	Mean FC 20 h versus 6 h	Meta logFC	Node type abbreviation	Cluster	Condition	Expression data type	SE	SD
TGL4	Triradylglycerols	1.25	1.35	NA	NA	NA	NA	Expressed in proteome	NA	NA
TGL4	Sterols	1.25	1.35	NA	NA	NA	NA	Expressed in proteome	NA	NA
FAS1	Fatty acids	1.04	0.96	NA	NA	NA	NA	Expressed in proteome	NA	NA
HFA1	Guanidines	NA	NA	NA	NA	NA	NA	No expression data	NA	NA
HMG1	Fatty acids	0.70	0.67	4.37	NHNB	Cluster 4	Lipid metabolism-coding genes	Expressed in proteome and transcriptome	0.158	0.223
FMS1	Amines	2.17	2.65	NA	NA	NA	NA	Expressed in proteome	NA	NA
ANB1	Amines	NA	NA	NA	NA	NA	NA	No expression data	NA	NA
LAT1	Fatty amides	1.37	1.51	2.74	HB	Cluster 9	Lipid metabolism-coding genes	Expressed in proteome and transcriptome	0.00817	0.0115
FMS1	Fatty amines	2.17	2.65	NA	NA	NA	NA	Expressed in proteome	NA	NA
SPO14	Cholines	0.65	0.91	NA	NA	NA	NA	Expressed in proteome	NA	NA

Feature	Associated molecules	Mean FC 12 h versus 6 h	Mean FC 20 h versus 6 h	Meta logFC	Node type abbreviation	Cluster	Condition	Expression data type	SE	SD
LCB5	Cholines	1.08	1.31	NA	NA	NA	NA	Expressed in proteome	NA	NA
CDC19	Short-chain acids	1.09	0.92	NA	NA	NA	NA	Expressed in proteome	NA	NA
CIT1	TCA acids	2.80	9.85	2.41	HB	Cluster 3	Lipid metabolism-coding genes	Expressed in proteome and transcriptome	0.258	0.366
FMS1	Carboxylic acids	2.17	2.65	NA	NA	NA	NA	Expressed in proteome	NA	NA
TOR1	Alcohols and polyols	0.74	0.58	NA	NA	NA	NA	Expressed in proteome	NA	NA
LSC2	Fatty amides	3.24	5.96	NA	NA	NA	NA	Expressed in proteome	NA	NA
LSC2	Fatty acids	3.24	5.96	NA	NA	NA	NA	Expressed in proteome	NA	NA
PDI1	Monosaccharides	1.27	1.20	2.93	NHB	Cluster 2	Proteostasis-coding genes	Expressed in proteome and transcriptome	0.112	0.159
ADH7	Monosaccharides	NA	NA	NA	NA	NA	NA	No expression data	NA	NA
GPP1	Monosaccharides	NA	NA	NA	NA	NA	NA	No expression data	NA	NA

Feature	Associated molecules	Mean FC 12 h versus 6 h	Mean FC 20 h versus 6 h	Meta logFC	Node type abbreviation	Cluster	Condition	Expression data type	SE	SD
YJU3	Monosaccharides	1.23	1.42	2.89	NHNB	Cluster 1	Lipid metabolism-coding genes	Expressed in proteome and transcriptome	0.315	0.446
SPO14	Monosaccharides	0.65	0.91	NA	NA	NA	NA	Expressed in proteome	NA	NA
GPD2	Monosaccharides	0.63	0.68	NA	NA	NA	NA	Expressed in proteome	NA	NA
HSP82	Monosaccharides	1.46	1.33	2.32	HB	Cluster 4	Proteostasis-coding genes	Expressed in proteome and transcriptome	0.0577	0.0816
ALD4	Fatty aldehydes	1.77	6.96	2.30	HB	Cluster 9	Lipid metabolism-coding genes	Expressed in proteome and transcriptome	0.0698	0.0988
LIP5	Fatty acids	0.90	1.14	NA	NA	NA	NA	Expressed in proteome	NA	NA
ALD4	Fatty acids	1.77	6.96	2.30	HB	Cluster 9	Lipid metabolism-coding genes	Expressed in proteome and transcriptome	0.0698	0.0988
FMS1	Amino acids and peptides	2.17	2.65	NA	NA	NA	NA	Expressed in proteome	NA	NA
GLT1	TCA acids	0.86	0.88	NA	NA	NA	NA	Expressed in proteome	NA	NA
LEU4	Fatty acids	1.01	1.64	NA	NA	NA	NA	Expressed in proteome	NA	NA

Feature	Associated molecules	Mean FC 12 h versus 6 h	Mean FC 20 h versus 6 h	Meta logFC	Node type abbreviation	Cluster	Condition	Expression data type	SE	SD
ACB1	NA	NA	NA	3.73	NA	NA	Lipid metabolism-coding genes	Expressed in transcriptome	0.0195	0.0275
ACC1	NA	NA	NA	3.14	NA	NA	Lipid metabolism-coding genes	Expressed in transcriptome	0.16	0.226
ACH1	NA	NA	NA	2.60	NA	NA	Lipid metabolism-coding genes	Expressed in transcriptome	0.0666	0.0941
ACL4	NA	NA	NA	2.50	NA	NA	Proteostasis-coding genes	Expressed in transcriptome	0.487	0.689
ACO1	NA	NA	NA	2.35	NA	NA	Lipid metabolism-coding genes	Expressed in transcriptome	0.0467	0.0661
ACP1	NA	NA	NA	3.43	NA	NA	Lipid metabolism-coding genes	Expressed in transcriptome	0.206	0.291
AFG3	NA	NA	NA	3.25	NA	NA	Proteostasis-coding genes	Expressed in transcriptome	0.0933	0.132
AHA1	NA	NA	NA	2.51	NA	NA	Proteostasis-coding genes	Expressed in transcriptome	0.0823	0.116
AIM45	NA	NA	NA	2.83	NA	NA	Lipid metabolism-coding genes	Expressed in transcriptome	0.253	0.358

Feature	Associated molecules	Mean FC 12 h versus 6 h	Mean FC 20 h versus 6 h	Meta logFC	Node type abbreviation	Cluster	Condition	Expression data type	SE	SD
ALD5	NA	NA	NA	2.52	NA	NA	Lipid metabolism-coding genes	Expressed in transcriptome	0.126	0.179
ALE1	NA	NA	NA	2.55	NA	NA	Lipid metabolism-coding genes	Expressed in transcriptome	0.0782	0.111
ALT1	NA	NA	NA	2.80	NA	NA	Lipid metabolism-coding genes	Expressed in transcriptome	0.000155	0.000219
APE1	NA	NA	NA	2.34	NA	NA	Autophagy-coding genes	Expressed in transcriptome	0.116	0.163
ARE1	NA	NA	NA	2.54	NA	NA	Lipid metabolism-coding genes	Expressed in transcriptome	0.257	0.363
ARG5,6	NA	NA	NA	3.09	NA	NA	Lipid metabolism-coding genes	Expressed in transcriptome	0.376	0.531
ARV1	NA	NA	NA	2.35	NA	NA	Lipid metabolism-coding genes	Expressed in transcriptome	0.345	0.488
ATG1	NA	NA	NA	2.30	NA	NA	Autophagy-coding genes	Expressed in transcriptome	0.177	0.251
ATG18	NA	NA	NA	2.43	NA	NA	Autophagy-coding genes	Expressed in transcriptome	0.341	0.482

Feature	Associated molecules	Mean FC 12 h versus 6 h	Mean FC 20 h versus 6 h	Meta logFC	Node type abbreviation	Cluster	Condition	Expression data type	SE	SD
ATG33	NA	NA	NA	2.35	NA	NA	Autophagy-coding genes	Expressed in transcriptome	0.0186	0.0263
ATG34	NA	NA	NA	3.34	NA	NA	Autophagy-coding genes	Expressed in transcriptome	0.407	0.576
ATG38	NA	NA	NA	2.35	NA	NA	Autophagy-coding genes	Expressed in transcriptome	0.00615	0.0087
ATG41	NA	NA	NA	2.99	NA	NA	Autophagy-coding genes	Expressed in transcriptome	0.338	0.478
ATG5	NA	NA	NA	2.13	NA	NA	Autophagy-coding genes	Expressed in transcriptome	0.036	0.0509
ATG8	NA	NA	NA	2.34	NA	NA	Autophagy-coding genes	Expressed in transcriptome	0.0856	0.121
BCS1	NA	NA	NA	2.43	NA	NA	Proteostasis-coding genes	Expressed in transcriptome	0.0985	0.139
BOL1	NA	NA	NA	2.04	NA	NA	Lipid metabolism-coding genes	Expressed in transcriptome	0.0009	0.00127
BOL3	NA	NA	NA	3.43	NA	NA	Lipid metabolism-coding genes	Expressed in transcriptome	0.194	0.275
CAT2	NA	NA	NA	2.97	NA	NA	Lipid metabolism-coding genes	Expressed in transcriptome	0.0832	0.118

Feature	Associated molecules	Mean FC 12 h versus 6 h	Mean FC 20 h versus 6 h	Meta logFC	Node type abbreviation	Cluster	Condition	Expression data type	SE	SD
CCP1	NA	NA	NA	2.67	NA	NA	Lipid metabolism-coding genes	Expressed in transcriptome	0.432	0.611
CCS1	NA	NA	NA	4.45	NA	NA	Proteostasis-coding genes	Expressed in transcriptome	0.63	0.891
CEM1	NA	NA	NA	3.66	NA	NA	Lipid metabolism-coding genes	Expressed in transcriptome	0.34	0.48
CHO1	NA	NA	NA	2.33	NA	NA	Lipid metabolism-coding genes	Expressed in transcriptome	0.233	0.329
CHO2	NA	NA	NA	2.50	NA	NA	Lipid metabolism-coding genes	Expressed in transcriptome	0.139	0.197
CIR1	NA	NA	NA	2.39	NA	NA	Lipid metabolism-coding genes	Expressed in transcriptome	0.078	0.11
COG2	NA	NA	NA	2.37	NA	NA	Autophagy-coding genes	Expressed in transcriptome	0.133	0.188
COX14	NA	NA	NA	2.24	NA	NA	Lipid metabolism-coding genes	Expressed in transcriptome	0.0864	0.122
CPR5	NA	NA	NA	5.24	NA	NA	Proteostasis-coding genes	Expressed in transcriptome	0.176	0.249

Feature	Associated molecules	Mean FC 12 h versus 6 h	Mean FC 20 h versus 6 h	Meta logFC	Node type abbreviation	Cluster	Condition	Expression data type	SE	SD
CPR6	NA	NA	NA	2.61	NA	NA	Proteostasis-coding genes	Expressed in transcriptome	0.0498	0.0704
CTA1	NA	NA	NA	3.27	NA	NA	Lipid metabolism-coding genes	Expressed in transcriptome	0.0669	0.0946
CUE5	NA	NA	NA	2.42	NA	NA	Autophagy-coding genes	Expressed in transcriptome	0.0471	0.0666
DAP1	NA	NA	NA	2.24	NA	NA	Lipid metabolism-coding genes	Expressed in transcriptome	0.154	0.218
DLD2	NA	NA	NA	2.37	NA	NA	Lipid metabolism-coding genes	Expressed in transcriptome	0.102	0.144
ECM10	NA	NA	NA	2.30	NA	NA	Proteostasis-coding genes	Expressed in transcriptome	0.111	0.158
ECM22	NA	NA	NA	2.44	NA	NA	Lipid metabolism-coding genes	Expressed in transcriptome	0.0416	0.0588
EDE1	NA	NA	NA	3.12	NA	NA	Proteostasis-coding genes	Expressed in transcriptome	0.0872	0.123
EEB1	NA	NA	NA	3.88	NA	NA	Lipid metabolism-coding genes	Expressed in transcriptome	0.119	0.168

Feature	Associated molecules	Mean FC 12 h versus 6 h	Mean FC 20 h versus 6 h	Meta logFC	Node type abbreviation	Cluster	Condition	Expression data type	SE	SD
ERG20	NA	NA	NA	2.14	NA	NA	Lipid metabolism-coding genes	Expressed in transcriptome	0.0161	0.0228
ERG25	NA	NA	NA	2.40	NA	NA	Lipid metabolism-coding genes	Expressed in transcriptome	0.0716	0.101
ERG28	NA	NA	NA	3.59	NA	NA	Lipid metabolism-coding genes	Expressed in transcriptome	0.0712	0.101
ERG29	NA	NA	NA	2.38	NA	NA	Lipid metabolism-coding genes	Expressed in transcriptome	0.0689	0.0974
ERG5	NA	NA	NA	2.48	NA	NA	Lipid metabolism-coding genes	Expressed in transcriptome	0.0882	0.125
ERG8	NA	NA	NA	4.56	NA	NA	Lipid metabolism-coding genes	Expressed in transcriptome	0.0168	0.0237
ERG9	NA	NA	NA	2.15	NA	NA	Lipid metabolism-coding genes	Expressed in transcriptome	0.0406	0.0574
ETR1	NA	NA	NA	2.24	NA	NA	Lipid metabolism-coding genes	Expressed in transcriptome	0.0564	0.0798

Feature	Associated molecules	Mean FC 12 h versus 6 h	Mean FC 20 h versus 6 h	Meta logFC	Node type abbreviation	Cluster	Condition	Expression data type	SE	SD
FAA2	NA	NA	NA	3.02	NA	NA	Lipid metabolism-coding genes	Expressed in transcriptome	0.355	0.502
FAS1	NA	NA	NA	2.52	NA	NA	Lipid metabolism-coding genes	Expressed in transcriptome	0.101	0.143
FAS2	NA	NA	NA	2.92	NA	NA	Lipid metabolism-coding genes	Expressed in transcriptome	0.123	0.175
FAT3	NA	NA	NA	3.56	NA	NA	Lipid metabolism-coding genes	Expressed in transcriptome	0.0902	0.128
FIS1	NA	NA	NA	4.39	NA	NA	Autophagy-coding genes	Expressed in transcriptome	0.346	0.489
FOX2	NA	NA	NA	2.26	NA	NA	Lipid metabolism-coding genes	Expressed in transcriptome	0.138	0.196
GCD7	NA	NA	NA	2.24	NA	NA	Autophagy-coding genes	Expressed in transcriptome	0.108	0.152
GCN4	NA	NA	NA	2.26	NA	NA	Autophagy-coding genes	Expressed in transcriptome	0.125	0.177
GET1	NA	NA	NA	2.42	NA	NA	Proteostasis-coding genes	Expressed in transcriptome	0.00709	0.01

Feature	Associated molecules	Mean FC 12 h versus 6 h	Mean FC 20 h versus 6 h	Meta logFC	Node type abbreviation	Cluster	Condition	Expression data type	SE	SD
GET2	NA	NA	NA	2.68	NA	NA	Proteostasis-coding genes	Expressed in transcriptome	0.0957	0.135
GET3	NA	NA	NA	3.67	NA	NA	Proteostasis-coding genes	Expressed in transcriptome	0.0541	0.0765
GET4	NA	NA	NA	2.65	NA	NA	Proteostasis-coding genes	Expressed in transcriptome	0.11	0.155
GLO4	NA	NA	NA	2.29	NA	NA	Lipid metabolism-coding genes	Expressed in transcriptome	0.201	0.285
GRS1	NA	NA	NA	2.84	NA	NA	Lipid metabolism-coding genes	Expressed in transcriptome	0.0346	0.0489
GUT1	NA	NA	NA	4.12	NA	NA	Lipid metabolism-coding genes	Expressed in transcriptome	0.189	0.267
HCH1	NA	NA	NA	2.95	NA	NA	Proteostasis-coding genes	Expressed in transcriptome	0.434	0.614
HFD1	NA	NA	NA	2.62	NA	NA	Lipid metabolism-coding genes	Expressed in transcriptome	0.0685	0.0969
HMG2	NA	NA	NA	2.19	NA	NA	Lipid metabolism-coding genes	Expressed in transcriptome	0.168	0.238

Feature	Associated molecules	Mean FC 12 h versus 6 h	Mean FC 20 h versus 6 h	Meta logFC	Node type abbreviation	Cluster	Condition	Expression data type	SE	SD
HSP104	NA	NA	NA	2.38	NA	NA	Proteostasis-coding genes	Expressed in transcriptome	0.0461	0.0652
HSP26	NA	NA	NA	4.44	NA	NA	Proteostasis-coding genes	Expressed in transcriptome	0.0231	0.0326
HSP42	NA	NA	NA	2.69	NA	NA	Proteostasis-coding genes	Expressed in transcriptome	0.0174	0.0247
HSP60	NA	NA	NA	2.49	NA	NA	Lipid metabolism-coding genes	Expressed in transcriptome	0.0922	0.13
HSP78	NA	NA	NA	2.16	NA	NA	Lipid metabolism-coding genes	Expressed in transcriptome	0.0538	0.076
HSV2	NA	NA	NA	2.19	NA	NA	Autophagy-coding genes	Expressed in transcriptome	0.189	0.267
ICL2	NA	NA	NA	2.62	NA	NA	Lipid metabolism-coding genes	Expressed in transcriptome	0.0434	0.0614
ICT1	NA	NA	NA	2.78	NA	NA	Lipid metabolism-coding genes	Expressed in transcriptome	0.194	0.275
IDH1	NA	NA	NA	2.44	NA	NA	Lipid metabolism-coding genes	Expressed in transcriptome	0.269	0.38

Feature	Associated molecules	Mean FC 12 h versus 6 h	Mean FC 20 h versus 6 h	Meta logFC	Node type abbreviation	Cluster	Condition	Expression data type	SE	SD
IDH2	NA	NA	NA	3.12	NA	NA	Lipid metabolism-coding genes	Expressed in transcriptome	0.0749	0.106
IDP3	NA	NA	NA	2.45	NA	NA	Lipid metabolism-coding genes	Expressed in transcriptome	0.109	0.155
IFA38	NA	NA	NA	2.35	NA	NA	Lipid metabolism-coding genes	Expressed in transcriptome	0.244	0.345
INO1	NA	NA	NA	2.76	NA	NA	Lipid metabolism-coding genes	Expressed in transcriptome	0.0952	0.135
IRC25	NA	NA	NA	2.37	NA	NA	Proteostasis-coding genes	Expressed in transcriptome	0.0668	0.0945
ISA2	NA	NA	NA	2.22	NA	NA	Lipid metabolism-coding genes	Expressed in transcriptome	0.0401	0.0567
ISM1	NA	NA	NA	2.21	NA	NA	Lipid metabolism-coding genes	Expressed in transcriptome	0.0972	0.137
ISU1	NA	NA	NA	3.20	NA	NA	Lipid metabolism-coding genes	Expressed in transcriptome	0.216	0.306

Feature	Associated molecules	Mean FC 12 h versus 6 h	Mean FC 20 h versus 6 h	Meta logFC	Node type abbreviation	Cluster	Condition	Expression data type	SE	SD
IZH2	NA	NA	NA	2.53	NA	NA	Lipid metabolism-coding genes	Expressed in transcriptome	0.252	0.356
IZH4	NA	NA	NA	3.08	NA	NA	Lipid metabolism-coding genes	Expressed in transcriptome	0.293	0.414
JAC1	NA	NA	NA	2.04	NA	NA	Lipid metabolism-coding genes	Expressed in transcriptome	0.0092	0.013
KAR2	NA	NA	NA	2.79	NA	NA	Proteostasis-coding genes	Expressed in transcriptome	0.172	0.243
MDH1	NA	NA	NA	2.28	NA	NA	Lipid metabolism-coding genes	Expressed in transcriptome	0.0452	0.0639
MDH3	NA	NA	NA	3.14	NA	NA	Lipid metabolism-coding genes	Expressed in transcriptome	0.0256	0.0362
MDJ1	NA	NA	NA	2.85	NA	NA	Lipid metabolism-coding genes	Expressed in transcriptome	0.0166	0.0235
MGA2	NA	NA	NA	2.49	NA	NA	Lipid metabolism-coding genes	Expressed in transcriptome	0.119	0.169

Feature	Associated molecules	Mean FC 12 h versus 6 h	Mean FC 20 h versus 6 h	Meta logFC	Node type abbreviation	Cluster	Condition	Expression data type	SE	SD
MGR2	NA	NA	NA	3.36	NA	NA	Proteostasis-coding genes	Expressed in transcriptome	0.448	0.633
MOT3	NA	NA	NA	2.52	NA	NA	Lipid metabolism-coding genes	Expressed in transcriptome	0.147	0.207
MSF1	NA	NA	NA	2.87	NA	NA	Lipid metabolism-coding genes	Expressed in transcriptome	0.0273	0.0385
MSS2	NA	NA	NA	2.57	NA	NA	Lipid metabolism-coding genes	Expressed in transcriptome	0.0524	0.0741
MZM1	NA	NA	NA	2.18	NA	NA	Lipid metabolism-coding genes	Expressed in transcriptome	0.115	0.162
NAP1	NA	NA	NA	5.06	NA	NA	Proteostasis-coding genes	Expressed in transcriptome	0.537	0.759
NCP1	NA	NA	NA	2.30	NA	NA	Lipid metabolism-coding genes	Expressed in transcriptome	0.0795	0.112
NDI1	NA	NA	NA	2.81	NA	NA	Lipid metabolism-coding genes	Expressed in transcriptome	0.0443	0.0627

Feature	Associated molecules	Mean FC 12 h versus 6 h	Mean FC 20 h versus 6 h	Meta logFC	Node type abbreviation	Cluster	Condition	Expression data type	SE	SD
OPI10	NA	NA	NA	3.46	NA	NA	Lipid metabolism-coding genes	Expressed in transcriptome	0.241	0.34
OPI3	NA	NA	NA	3.49	NA	NA	Lipid metabolism-coding genes	Expressed in transcriptome	2.98e-05	4.21e-05
ORM2	NA	NA	NA	4.12	NA	NA	Proteostasis-coding genes	Expressed in transcriptome	0.185	0.261
OTU1	NA	NA	NA	2.37	NA	NA	Proteostasis-coding genes	Expressed in transcriptome	0.132	0.186
PAM16	NA	NA	NA	2.17	NA	NA	Proteostasis-coding genes	Expressed in transcriptome	0.136	0.192
PAM18	NA	NA	NA	2.25	NA	NA	Proteostasis-coding genes	Expressed in transcriptome	0.0668	0.0944
PCD1	NA	NA	NA	2.36	NA	NA	Lipid metabolism-coding genes	Expressed in transcriptome	0.283	0.4
PDA1	NA	NA	NA	2.55	NA	NA	Lipid metabolism-coding genes	Expressed in transcriptome	0.0786	0.111
PDB1	NA	NA	NA	2.96	NA	NA	Lipid metabolism-coding genes	Expressed in transcriptome	0.0999	0.141

Feature	Associated molecules	Mean FC 12 h versus 6 h	Mean FC 20 h versus 6 h	Meta logFC	Node type abbreviation	Cluster	Condition	Expression data type	SE	SD
PEP4	NA	NA	NA	3.93	NA	NA	Autophagy-coding genes	Expressed in transcriptome	0.0828	0.117
PET100	NA	NA	NA	2.20	NA	NA	Proteostasis-coding genes	Expressed in transcriptome	0.123	0.174
PHB1	NA	NA	NA	2.24	NA	NA	Proteostasis-coding genes	Expressed in transcriptome	0.176	0.248
PHB2	NA	NA	NA	3.21	NA	NA	Proteostasis-coding genes	Expressed in transcriptome	0.187	0.265
PHO86	NA	NA	NA	2.23	NA	NA	Proteostasis-coding genes	Expressed in transcriptome	0.0863	0.122
PIM1	NA	NA	NA	2.24	NA	NA	Lipid metabolism-coding genes	Expressed in transcriptome	0.0631	0.0893
PKP1	NA	NA	NA	2.80	NA	NA	Lipid metabolism-coding genes	Expressed in transcriptome	0.0812	0.115
PLB2	NA	NA	NA	2.07	NA	NA	Lipid metabolism-coding genes	Expressed in transcriptome	0.021	0.0297
PMT1	NA	NA	NA	2.53	NA	NA	Proteostasis-coding genes	Expressed in transcriptome	0.048	0.0679
PPX1	NA	NA	NA	2.45	NA	NA	Lipid metabolism-coding genes	Expressed in transcriptome	0.0689	0.0974

Feature	Associated molecules	Mean FC 12 h versus 6 h	Mean FC 20 h versus 6 h	Meta logFC	Node type abbreviation	Cluster	Condition	Expression data type	SE	SD
PTC2	NA	NA	NA	3.56	NA	NA	Proteostasis-coding genes	Expressed in transcriptome	1.33	1.88
PUT1	NA	NA	NA	3.22	NA	NA	Lipid metabolism-coding genes	Expressed in transcriptome	0.219	0.31
PXA1	NA	NA	NA	2.35	NA	NA	Lipid metabolism-coding genes	Expressed in transcriptome	0.122	0.173
PXA2	NA	NA	NA	2.29	NA	NA	Lipid metabolism-coding genes	Expressed in transcriptome	0.074	0.105
RPL5	NA	NA	NA	3.28	NA	NA	Proteostasis-coding genes	Expressed in transcriptome	0.331	0.468
RRI1	NA	NA	NA	2.14	NA	NA	Lipid metabolism-coding genes	Expressed in transcriptome	0.00375	0.0053
RTN1	NA	NA	NA	2.87	NA	NA	Proteostasis-coding genes	Expressed in transcriptome	0.0882	0.125
RTT106	NA	NA	NA	2.17	NA	NA	Proteostasis-coding genes	Expressed in transcriptome	0.0565	0.0799
SAH1	NA	NA	NA	4.06	NA	NA	Lipid metabolism-coding genes	Expressed in transcriptome	0.409	0.579

Feature	Associated molecules	Mean FC 12 h versus 6 h	Mean FC 20 h versus 6 h	Meta logFC	Node type abbreviation	Cluster	Condition	Expression data type	SE	SD
SBA1	NA	NA	NA	6.04	NA	NA	Proteostasis-coding genes	Expressed in transcriptome	0.299	0.423
SCS3	NA	NA	NA	2.59	NA	NA	Lipid metabolism-coding genes	Expressed in transcriptome	0.0104	0.0146
SEC17	NA	NA	NA	3.78	NA	NA	Autophagy-coding genes	Expressed in transcriptome	0.00623	0.00882
SEC18	NA	NA	NA	2.44	NA	NA	Lipid metabolism-coding genes	Expressed in transcriptome	0.0791	0.112
SEC4	NA	NA	NA	3.06	NA	NA	Autophagy-coding genes	Expressed in transcriptome	0.0109	0.0154
SEC7	NA	NA	NA	3.18	NA	NA	Autophagy-coding genes	Expressed in transcriptome	0.0293	0.0414
SGT2	NA	NA	NA	2.12	NA	NA	Proteostasis-coding genes	Expressed in transcriptome	0.0145	0.0204
SHQ1	NA	NA	NA	2.09	NA	NA	Proteostasis-coding genes	Expressed in transcriptome	0.054	0.0763
SNO4	NA	NA	NA	2.98	NA	NA	Proteostasis-coding genes	Expressed in transcriptome	0.0136	0.0192
SOD2	NA	NA	NA	2.19	NA	NA	Lipid metabolism-coding genes	Expressed in transcriptome	0.0085	0.012

Feature	Associated molecules	Mean FC 12 h versus 6 h	Mean FC 20 h versus 6 h	Meta logFC	Node type abbreviation	Cluster	Condition	Expression data type	SE	SD
SPT4	NA	NA	NA	2.17	NA	NA	Autophagy-coding genes	Expressed in transcriptome	0.111	0.156
SSA1	NA	NA	NA	3.60	NA	NA	Proteostasis-coding genes	Expressed in transcriptome	0.346	0.489
SSA2	NA	NA	NA	2.53	NA	NA	Proteostasis-coding genes	Expressed in transcriptome	0.158	0.223
SSA3	NA	NA	NA	3.93	NA	NA	Proteostasis-coding genes	Expressed in transcriptome	0.302	0.428
SSA4	NA	NA	NA	4.28	NA	NA	Proteostasis-coding genes	Expressed in transcriptome	0.388	0.548
SSB2	NA	NA	NA	2.70	NA	NA	Proteostasis-coding genes	Expressed in transcriptome	0.0218	0.0309
SSZ1	NA	NA	NA	2.93	NA	NA	Proteostasis-coding genes	Expressed in transcriptome	0.0404	0.0571
SUV3	NA	NA	NA	3.71	NA	NA	Lipid metabolism-coding genes	Expressed in transcriptome	1.09	1.55
SYT1	NA	NA	NA	2.65	NA	NA	Proteostasis-coding genes	Expressed in transcriptome	0.0187	0.0264
TAM41	NA	NA	NA	2.31	NA	NA	Lipid metabolism-coding genes	Expressed in transcriptome	0.114	0.161

Feature	Associated molecules	Mean FC 12 h versus 6 h	Mean FC 20 h versus 6 h	Meta logFC	Node type abbreviation	Cluster	Condition	Expression data type	SE	SD
TAZ1	NA	NA	NA	2.27	NA	NA	Lipid metabolism-coding genes	Expressed in transcriptome	0.16	0.226
TES1	NA	NA	NA	2.31	NA	NA	Lipid metabolism-coding genes	Expressed in transcriptome	0.145	0.205
TIM12	NA	NA	NA	2.71	NA	NA	Proteostasis-coding genes	Expressed in transcriptome	0.0359	0.0508
TIM17	NA	NA	NA	2.35	NA	NA	Proteostasis-coding genes	Expressed in transcriptome	0.18	0.254
TIM54	NA	NA	NA	2.62	NA	NA	Proteostasis-coding genes	Expressed in transcriptome	0.186	0.263
TIM8	NA	NA	NA	2.16	NA	NA	Proteostasis-coding genes	Expressed in transcriptome	0.0785	0.111
TIM9	NA	NA	NA	3.57	NA	NA	Proteostasis-coding genes	Expressed in transcriptome	0.00922	0.013
TMA17	NA	NA	NA	4.42	NA	NA	Proteostasis-coding genes	Expressed in transcriptome	0.023	0.0326
TML25	NA	NA	NA	3.23	NA	NA	Lipid metabolism-coding genes	Expressed in transcriptome	0.36	0.509
TOM6	NA	NA	NA	2.80	NA	NA	Proteostasis-coding genes	Expressed in transcriptome	0.0598	0.0846

Feature	Associated molecules	Mean FC 12 h versus 6 h	Mean FC 20 h versus 6 h	Meta logFC	Node type abbreviation	Cluster	Condition	Expression data type	SE	SD
TOM7	NA	NA	NA	2.23	NA	NA	Proteostasis-coding genes	Expressed in transcriptome	0.032	0.0452
TRS120	NA	NA	NA	2.60	NA	NA	Autophagy-coding genes	Expressed in transcriptome	0.0276	0.039
TSA1	NA	NA	NA	2.52	NA	NA	Proteostasis-coding genes	Expressed in transcriptome	0.0385	0.0544
TSC3	NA	NA	NA	3.44	NA	NA	Lipid metabolism-coding genes	Expressed in transcriptome	0.822	1.16
UMP1	NA	NA	NA	2.80	NA	NA	Proteostasis-coding genes	Expressed in transcriptome	0.217	0.306
UPS1	NA	NA	NA	3.53	NA	NA	Lipid metabolism-coding genes	Expressed in transcriptome	0.176	0.249
UPS2	NA	NA	NA	3.54	NA	NA	Lipid metabolism-coding genes	Expressed in transcriptome	0.361	0.511
VPS75	NA	NA	NA	2.10	NA	NA	Proteostasis-coding genes	Expressed in transcriptome	0.00781	0.011
WHI2	NA	NA	NA	3.51	NA	NA	Autophagy-coding genes	Expressed in transcriptome	0.889	1.26
YAP1	NA	NA	NA	2.15	NA	NA	Proteostasis-coding genes	Expressed in transcriptome	0.109	0.154

Feature	Associated molecules	Mean FC 12 h versus 6 h	Mean FC 20 h versus 6 h	Meta logFC	Node type abbreviation	Cluster	Condition	Expression data type	SE	SD
YDC1	NA	NA	NA	2.82	NA	NA	Lipid metabolism-coding genes	Expressed in transcriptome	0.0118	0.0167
YFH1	NA	NA	NA	2.63	NA	NA	Lipid metabolism-coding genes	Expressed in transcriptome	0.409	0.578
YHB1	NA	NA	NA	3.39	NA	NA	Lipid metabolism-coding genes	Expressed in transcriptome	0.031	0.0438
YME1	NA	NA	NA	3.03	NA	NA	Proteostasis-coding genes	Expressed in transcriptome	0.0967	0.137
YOS9	NA	NA	NA	2.22	NA	NA	Proteostasis-coding genes	Expressed in transcriptome	0.174	0.247
YPT1	NA	NA	NA	3.31	NA	NA	Autophagy-coding genes	Expressed in transcriptome	0.153	0.217
YPT31	NA	NA	NA	4.07	NA	NA	Autophagy-coding genes	Expressed in transcriptome	0.145	0.205
YPT52	NA	NA	NA	2.24	NA	NA	Autophagy-coding genes	Expressed in transcriptome	0.118	0.168
YPT6	NA	NA	NA	2.29	NA	NA	Autophagy-coding genes	Expressed in transcriptome	0.172	0.243

Feature	Associated molecules	Mean FC 12 h versus 6 h	Mean FC 20 h versus 6 h	Meta logFC	Node type abbreviation	Cluster	Condition	Expression data type	SE	SD
ZIM17	NA	NA	NA	2.75	NA	NA	Lipid metabolism-coding genes	Expressed in transcriptome	0.437	0.618
